# Gelatin-Polyvinyl Alcohol Film for Tissue Engineering: A Concise Review

**DOI:** 10.3390/biomedicines9080979

**Published:** 2021-08-09

**Authors:** Izzat Zulkiflee, Mh Busra Fauzi

**Affiliations:** Centre for Tissue Engineering and Regenerative Medicine, Faculty of Medicine, Universiti Kebangsaan Malaysia, Jalan Yaacob Latiff, Bandar Tun Razak, Kuala Lumpur 56000, Malaysia; mizzatzulkiflee@gmail.com

**Keywords:** gelatin, PVA, film, scaffold, tissue engineering

## Abstract

The field of biomaterials has been steadily expanding as a large number of pharmaceutical and manufacturing companies invest in research in order to commercialize biomaterial products. Various three-dimensional biomaterials have been explored including film, hydrogel, sponge, microspheres etc., depending on different applications. Thus, gelatin and polyvinyl alcohol (PVA) are widely used as a natural- and synthetic-based biomaterial, respectively, for tissue engineering and clinical settings. The combination of these materials has proven its synergistic effects in wound-healing applications. Therefore, this review aims to highlight the hybrid gelatin and PVA thin film development and evaluate its potential characteristics for tissue engineering applications from existing published evidence (within year 2010–2020). The primary key factor for polymers mixing technology might improve the quality and the efficacy of the intended polymers. This review provides a concise overview of the current knowledge for hybrid gelatin and PVA with the method of fabricating and mixing technology into thin films. Additionally, the findings guided to an optimal fabrication method and scrutinised characterisation parameters of fabricated gelatin-PVA thin film. In conclusion, hybrid gelatin-PVA thin film has higher potential as a treatment for various biomedical and clinical applications.

## 1. Introduction

### 1.1. Tissue Engineering

**Figure 1:**
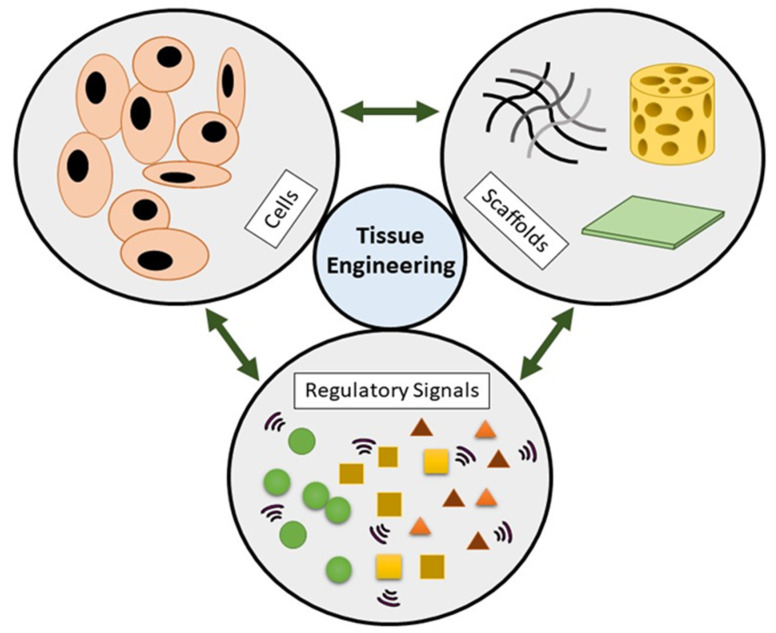
Three main components of tissue engineering.

The tissue engineering (TE) field is an advanced research discipline primarily focusing on producing tissues and organ replacements by regulating cellular and biomechanical parameters in the laboratory. TE triad includes the cells as a tissue-building unit whereby bioscaffold acts as a platform for the cells to grow creating solid tissue form and the biomolecules component act as enhancer or supplement. Figure 1 shows the three essential components in TE. The exponential development of biomaterial technology has revolutionised its use in the biological and industrial fields over the past few decades [1]. Given their widely used applications as bioequivalent materials, polymer blends are very significant and belong to a rapidly advanced branch of polymer science and technology as well as medical applications. In the medical field, polymer blends are used in acute wounds namely burn, trauma, radiation and surgery or chronic diseases such as diabetes, obesity and ulcers (pressure ulcers) or delayed acute wound healing [2]. In the interests of the quality of patient care and medical research, this will revolutionise medicine towards the various field of drug delivery, drug resistance, gene therapy, diagnostics, medical therapies, treatment protocols, immunomodulators or simulants therapy, surgical interventions and related research areas [3].

Polymer mixing is a frequently used approach in polymer research to create polymers with better characteristics. Polymer mixtures (solutions) are often viscous, and the time taken to achieve phase separation might be substantially greater than the equivalent solutions containing tiny nonpolymeric molecules to dissolve [4]. In addition, polymer mixing has been reported to include a massive effect on the physical properties including the mechanical, thermal, optical and electrical properties [5]. Once these polymers are adequately mixed with an appropriate substrate, they can interfere either in the amorphous or crystalline fraction of the polymers, hence altering the polymeric properties. Such changes in the physical properties depend on the composition of the guest material chemical composition and how it interacts with the host polymer.

### 1.2. Composite/Hybrid Biomaterials

Bioscaffolds are made using a combination of lab and industrial-scale processes, and the final product must have specific properties to make them ideal for TE applications. Fabrication of natural materials into scaffolds that preserve the natural materials’ original bioactivity and functionality remains a challenge [6]. The scaffolds need to mimic or resemble the 3D microstructure of the native extracellular matrix (ECM) architecture [7]. ECM is highly responsible for many cell functions including cell assembly into tissue and organs, cell-to-cell interactions and growth regulation [8]. There are many forms of bioscaffolds in TE and this review focuses on the blended film’s exploration.

Composite materials are 3D-designed materials made up of two or more constituent materials with prominently different physical, chemical and mechanical characteristics. This review discusses a combination of gelatin and PVA materials due to their biocompatibility, versatility and biodegradability properties. Gelatin and PVA have excellent film-forming properties and can be potentially applied to various TE, biomedical and clinical settings. The major disadvantage of gelatin-based materials is poor mechanical qualities, thermally unstable and faster degradation rate [9]. The mechanical efficiency and environmental stability of the composite materials are largely determined by the interface between matrix and reinforcement [10]. The mixture of gelatin and PVA may help to improve its mechanical properties and muco-adhesiveness, especially in the film form. The composite/hybrid biomaterials could reduce the failure of implanted materials at the wound site and sustain the optimum microenvironment primarily in wet condition that improves the wound-healing mechanism. A tunable hybrid thin film might be useful in modulating different cell behaviour of a tissue type and this will expedite the wound closure compared to without any treatment. The synergistic effects of hybrid biomaterials such as gelatin/PVA could influence the common wound-healing phases including inflammatory, proliferative and remodelling phases.

### 1.3. Film Applications and Its Advantage

Looking at the unique properties of the films, a 3D scaffold fabricated by casting and air-dried has been applied in TE such as drug delivery, wound dressing and medical applications. In wound dressing, 3D-bioscaffold needs to be non-toxic, allow for gas exchanges, be able to absorb wound exudates and help in protecting the wound from any microbial organisms [11]. Films may be different compared to other types of scaffolds such as porous scaffolds, hydrogels, nanofibers or even nanoparticles. However, 3D-shaped scaffolds such as films offer additional options such as expanding the range of structures accessible to address skin repair complications. Gelatin-PVA film being a bio-degradable scaffold can be another alternative product to replace wound dressings in the wound-healing treatment. A dressing can be of high cost due to an immense amount of re-dressing on a wound to prevent antibacterial infection, abrasions and ulcers. A film is easily fabricated and can be immediately used on different kinds of wounds. 

**Figure 2:**
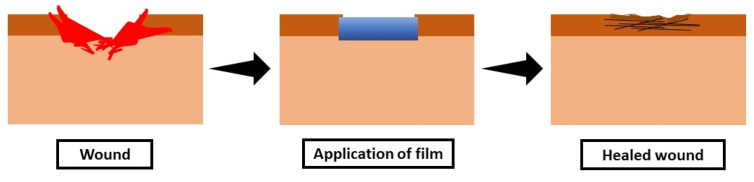
Application of film on skin wound.

Compared to other types of scaffolds, sponge porous scaffolds may have situations such as collapsed scaffolds due to extensive movements during the application on the wounds or limited control of pore structure on the electrospun scaffolds. With the help of PVA, the gelatin-PVA film has high tensile strength and flexibility to improve its durability during application. The gelatin-PVA film will provide a strong base as a scaffold with the additional or mixing of many other biomaterials that can help in many kinds of applications including wound healing. Gelatin-PVA film is considered a biological dressing that is capable to incorporate with other materials to promote the wound-healing process by stimulating the responses of the cells [12,13,14]. In wound healing, it is important to acknowledge the stages of wound healing to improve the understanding and the importance of the application of the film on wounds. The three main key features for wound-healing applications of an ideal scaffold are: good physical and mechanical capabilities, as well as an excellent physiological background to facilitate cell adhesion and proliferation and/or differentiation [15]. Even though the healing process is continual, it can be divided into four distinct phases: haemostasis, inflammation, proliferation or granulation and remodelling or maturation [16]. Yet, skin regeneration and wound healing can be delayed, resulting in persistent inflammation, particularly in burn victims. In addition, a scaffold that can control the moisture and absorbs exudates from the wound such as film can improve wound-healing treatments. Figure 2 displays the idea of the application of film on wound for wound-healing treatment.

### 1.4. Biomaterials for the Film

Biomaterials are usually classified into two main groups, which are natural and synthetics. Natural biomaterials are usually sourced from various natural products from mostly animals or plants, grouped into protein-based biomaterials, polysaccharide-based biomaterials and others. Natural biomaterials are usually needed for clinical use due to their excellent biocompatibility, biodegradability, functionality and low immunogenicity [17]. On the other hand, synthetic biomaterials can be classified as polymers that are bio-degradable or non-bio-degradable including metals and ceramics.

Natural biomaterials are massively explored from green resources since it involves an affordable cost in upscaling and most likely biocompatible with other living things. It is also bio-degradable and resorbable with very low toxicity [18] and low inflammatory response and biofunctionalisation. Due to their benefits, these materials are typically used to repair or rebuild the structure and function of weakened or damaged tissues or organs. In addition, they can provide sufficient support for cell adhesion, migration, proliferation and differentiation [19]. However, the cost of using natural biomaterials can be high for certain products and with limited resources followed by being life-threatening to the ecosystem. Other than that, natural-based biomaterials possess certain drawbacks due to their poor mechanical strength without appropriate crosslinkers. These natural polymers are able to crosslink or self-assemble to create a non-cytotoxic bioscaffold resembling the ECM and other physicochemical properties of native tissue [20]. Crosslinkers are supporting components helping the biomaterials to retain their mechanical strengths [21], as well as enhancing the stability [22] and complex structural assembly of the fabricated biomaterials [23]. 

In addition, the synthetic biomaterial requires many tweaking and modifications to look like or mimic the natural ECM. It involves the use of binding ligands, biological signals and cell biocompatibility [24]. Some of the materials can be cost-effective and the sources can be easily obtained commercially. It can be used to replace natural biomaterials for tissue engineering and regenerative medicine. Each scaffold should provide biomaterial composition, mechanical strength and architecture properties which will determine the effectiveness of the cellular interaction, functional effectiveness in tissue regeneration as well as the successful integration of the host tissues [25]. Some synthetic biomaterials are bio-degradable and the degradation products are usually used as temporary implants or delivery systems [26]. Synthetic and natural polymers have been widely applied and used as biomaterial to fabricate scaffolds to accomplish these properties.

**Figure 3:**
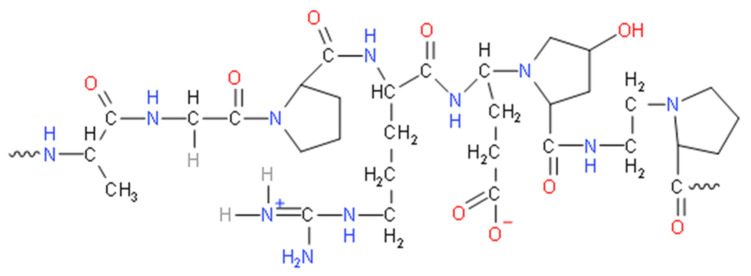
The chemical structure of gelatin.

**Figure 4:**
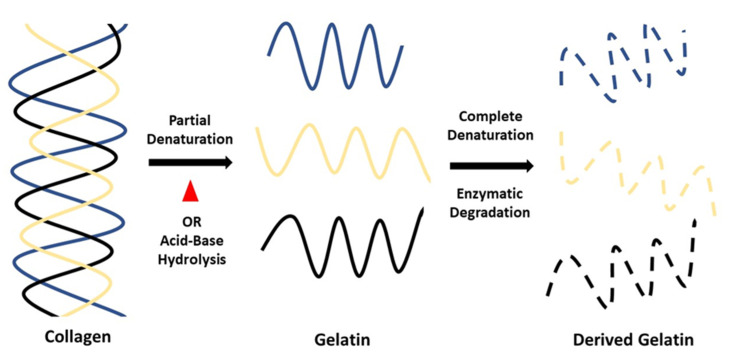
Process of denaturation of collagen into gelatin.

Gelatin is one of the natural polymers that has been widely used in many fields, especially medical and scientific applications. Gelatin is a polymer formed by partial hydrolysis of skin-derived collagen, white connective tissues and animal bones [27,28,29,30]. It is made by irreversibly hydrolysing collagen’s triple helical structure, resulting in random coiled domains using heat and enzymatic denaturation [31]. As a result, the derived gelatin can be applied for the fabrication of scaffolds. Figure 3 shows the chemical structure of gelatin while Figure 4 shows a simple process of denaturation of collagen into gelatin. As a result, the gelatin is less organised than collagen but has a relatively similar molecular composition. There are two types of gelatin: type A (derived via partial acid) and type B (derived via alkaline hydrolysis) [28]. Different sources and manufacturing procedures lead to various physical properties and chemical heterogeneity [32]. Basic gelatin is often used to deliver acidic bioactive agents while acidic gelatin is used to deliver basic bioactive agents [33]. Gelatin type A has an isoelectric point approximately around pH 7 to pH 9 while gelatin type B has a pH value approximately around 4.8 to 5.1 [29,32]. As a protein, it is used in food, cosmetics, pharmaceuticals and photographic industries for its gel-forming, non-toxic and low cost of production. It is known for its biocompatibility, bio-degradability, low immunogenicity and high resorbability with certain limitations such as low mechanical strength and sensitivity to heat [34,35,36,37]. For pharmaceuticals, gelatin is commonly used as a shell capsule to control raw materials processing [38]. Gelatin is also widely used in advanced technology as a bioink for bioprinting due to its good fidelity, modifiable material, biocompatibility, degradability and rheological properties [39]. Besides, gelatin is widely studied in bio-degradable film development and characterisation studies as pure and blended with other biopolymers due to its excellent film-forming properties [40].

**Figure 5:**
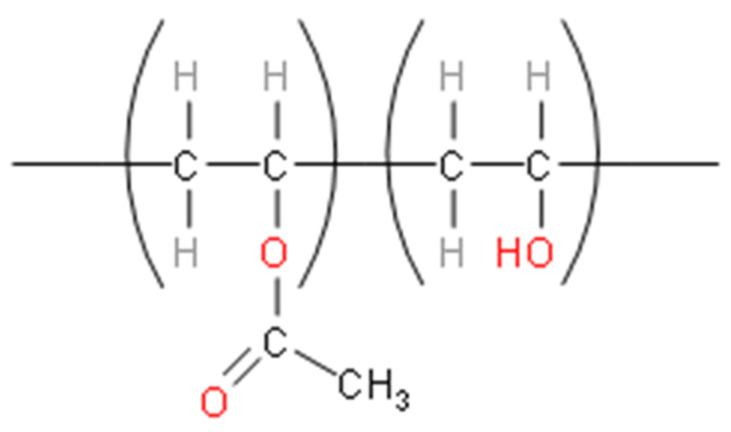
Chemical structure of PVA.

PVA, a synthetic polymer that is approved by FDA can be used as an alternative for polar and water-soluble material. It has been established as bio-degradable synthetic polymers with hydrophilic nature characteristics [41]. It is a flexible polymer and the only synthesised polymer with a backbone largely made up of –OH bonds that are fully bio-degradable. The bio-degradability of PVA is ultimately determined by its degree of hydrolysis and molecular weight that are potentially used for biological applications. PVA has good film formation capability, solid conglutination and excellent thermal stability [42]. PVA is also known as a “green polymer” because of its solubility trend and ease of degradability [43]. PVA can be chemically crosslinked or stabilised using physical entanglement to be used in medical and pharmaceutical applications to overcome the ageing effect [28]. Other than that, PVA is commonly blended with other compounds to help improve the mechanical properties due to its hydrophilic properties and compatible structure [44]. In this research, gelatin and PVA are selected as the primary materials for preparing a new hybrid bio-degradable film using additional monomers and modification via irradiation intervention. Figure 5 shows the chemical structure of polyvinyl alcohol.

Both gelatin and PVA do not possess any antioxidant and antimicrobial properties unless the fabricated scaffold or film is incorporated with other materials that can improve its antioxidant and antimicrobial properties. Many studies include other materials as an additional bioactive or biomaterial to help improve the scaffold’s properties. For example, a mixture of PVA with basil leaf extract helps increase the antioxidant and antimicrobial properties of the film [45]. Some other materials that have been studied to improve the antibacterial properties of the film include tomato pulp [46], zinc oxide nanoparticles [47], bacterial cellulose nanowhiskers [48] and ciprofloxacin hydrochloride [49]. These show that gelatin and PVA can be mixed or incorporated together with many other materials to improve not only the antioxidant properties but also antimicrobial properties. Besides, the gelatin and PVA thin film could be coated via plasma polymerisation with antibacterial or antioxidant active compounds. However, the selection of a polymerisable candidate from a specific active compound is carried out depending on its characteristics such as being highly volatile to actively react with the plasma approach to coat the surfaces of a particular film.

## 2. Literature Search and Data Extraction Management

**Figure 6:**
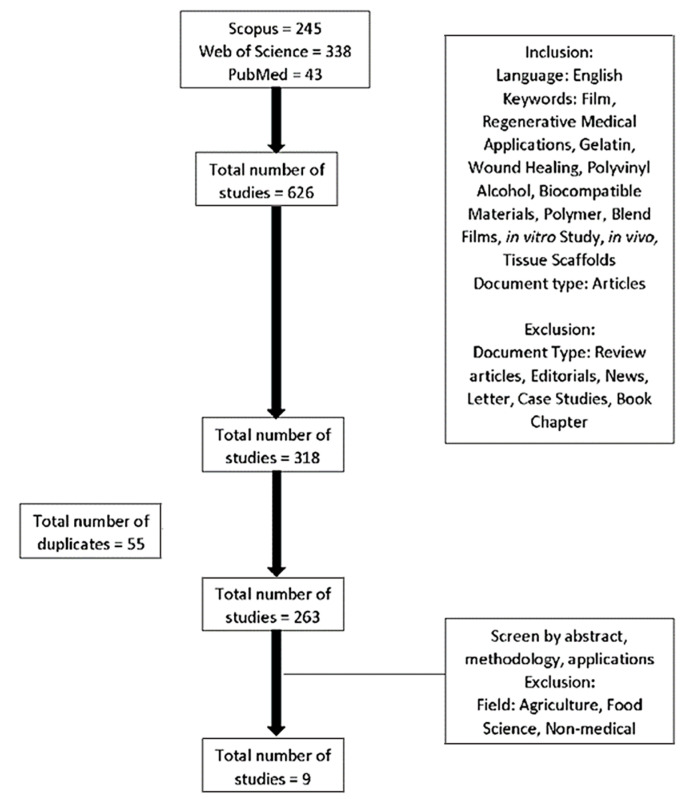
Flowchart of article selection and data extraction management.

A few databases were searched using specific English keywords from 2010 to 2021 with inevitable inclusion and exclusion criteria to select related articles. The keywords search yielded 626 articles, of which 9 specific articles were selected to be examined in this review. The fabrication methods, the ratio of gelatin to PVA, type of crosslinkers and certain parameters for the characterisation of the fabricated films were discussed. Figure 6 demonstrates the articles’ selection and data extraction management. Our review shows that film from gelatin and PVA mixture can be a good film and suitable for medical applications, especially in tissue engineering. The exclusion criteria for this review were all secondary literature and any original articles that have been written and submitted in different languages other than English.

## 3. Fabrication of Gelatin-PVA Film

In this section, we reported the selected studies based on their fabrication methods. A thorough discussion is carried out in the discussion section. Based on the previous studies, there are many different concentrations that researchers have used for the designated experimental procedures. Most of the studies did not mention the gelatin and PVA concentrations that are prominently challenging to provide an optimum ratio in thin-film fabrication. As for gelatin, the final concentrations used by Chaibi et al. [50] and Ismaiel et al. [51] are almost similar, which are 0.05 and 0.048 g/mL, respectively. In contrast, Al-Mamun et al. [52] used a higher concentration of 0.11 g/mL. Another study performed by Basak et al. [53] described 5% of gelatin, however, Jain et al. [54] used three different percentages of gelatin that are 1%, 2% and 3%. The PVA concentration in Al-Mamun et al. [52] and Ismaiel [51] was 0.11 and 0.032 g/mL, respectively for their blended gelatin-PVA. Furthermore, Chaibi [50] scrutinised that PVA has been used around 0.2% by weight/volume. Basak [53] and Jain [54] research reported that they used 5% and 10% PVA, respectively. The blended approach needs the best ratio of gelatin and PVA to fabricate a suitable film for different applications. Literature search demonstrated a various ratio of gelatin to PVA that has been reported in the selected articles. Al-Mamun et al. [52] and Ebnalwaled et al. [55] reported a 1:1 ratio of gelatin to PVA. In contrast, Basak et al. [53] and Chaibi et al. [50] used fixed ratios, which are 1:2 and 29:43, respectively. The remaining studies from other articles had various ratios per study for their optimisation purposes. The list of the concentrations, ratios, fabrication method and molecular weight of PVA from all studies is tabulated in Table 1.

Some reports omitted the use of any crosslinker to crosslink the gelatin and PVA, however, there are some studies that use crosslinker to strengthen the link between gelatin and PVA. The most common crosslinkers stated in the studies include glutaraldehyde and gamma-irradiation. The crosslinking methods were either pre-crosslinking (addition of glutaraldehyde during mixing) or post-crosslinking (gamma-irradiated or soaked in glutaraldehyde solution). Detailed reasoning on the use of these crosslinkers is further discussed in the discussion section.

**Figure 7:**
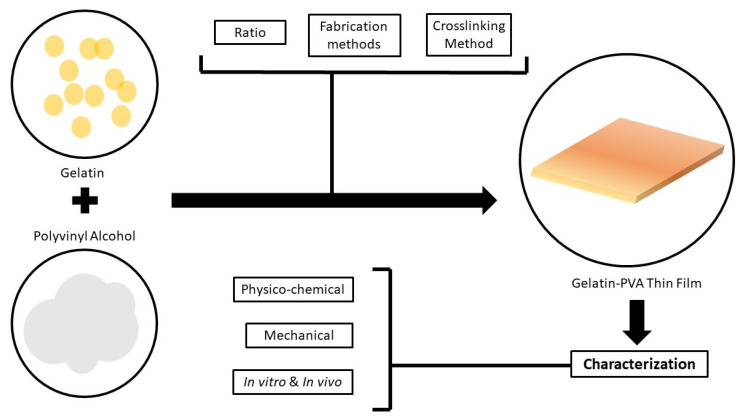
Flow of method for fabrication and characterisation of the film.

Basak et al. [53] and Chaibi et al. [50] used glutaraldehyde as a crosslinker while Al-Mamun et al. [52], Ismaiel et al. [51], Ebnalwaled et al. [55] and Khan et al. [56] reported the use of gamma-irradiation as a method of crosslinking. While there are some studies that did not use any crosslinkers in their fabrication methods. For the fabrication technique, most articles presented a similar approach whereby gelatin and PVA were either prepared in different solutions and then added together or mixed by powder at different temperatures (mostly 40 °C and above). The solutions were then casted into a mould or a glass plate and left to dry. A different method was reported in which the film was casted into a plexiglass plate, dried and heated at 500 °C in an oven [51] for 12 h. Basak et al. [53] reported that the film was casted at 40 °C for 24 h for the drying process. The other studies performed the same method of drying whereby the solutions were left to dry at room temperature. Figure 7 shows the flow of the fabrication method and characterisation of the film. 

## 4. Gelatin-PVA Film Characterisation

In this section, the only report on the film characterisations was based on a few parameters involved taken from the selected articles. An extensive explanation is discussed in the discussion section to provide prominent output and key points to support the current findings. Table 2 demonstrates the characterisation of gelatin-PVA film.

### 4.1. Fourier Transform Infrared (FTIR) Analysis

Firstly, the FTIR findings on the blended films crosslinked with gamma-irradiation were reported. Al-Mamun et al. [52] demonstrated that the presence of a shoulder shape occurred at 2900 cm^−1^ and 3300 cm^−1^. The peaks seem clearly visible and prominent compared to other samples with a higher dose of gamma-irradiation. This phenomenon is similarly reported by Ismaeil et al. [51] in which the films with a higher irradiation dosage described more prominent peaks than the non-irradiated films. Ebnalwaled et al. [55] reported on the mixture of gelatin and PVA with CuO. Increasing the dosage of gamma irradiation increases the intensity of the peaks followed by an increased gelatin concentration. Finally, Khan et al. [56] reported that grafted film does not show any peak characteristic corresponding to the carbonyl group and amino group, hence indicating a complete crosslinking through these functional groups.

Secondly, glutaraldehyde has been used as a crosslinker to enhance the blended films as stated in the selected articles. Basak et al. [53] mentioned that the interfacial interaction between PVA-gelatin/HAP composite was confirmed based on the peak compared to the pure peak of HAP. Additionally, Chaibi et al. [50] reported that physical crosslinking bands were shown between gelatin and PVA via glutaraldehyde intervention. Thus, adding glutaraldehyde to the blend proved the success of chemical crosslinking. In contrast, El-Bahy et al. [57] stated no new bands were formed, which shows an absence of chemical interaction between gelatin and PVA in their experiment. Nonetheless, Jain et al. [54] confirmed that complete esterification of the carboxylic acid of gelatin and PVA indicate the occurrence of chemical interaction between gelatin and PVA.

### 4.2. Mechanical Properties

Tensile strength (TS) as reported by Al-Mamun et al. [52] revealed that TS increased with an increase in radiation. However, there was no significant difference between crosslinked and non-crosslinked films from their findings. The percentage of elongation at break (Eb) decreased with the increase of radiation doses. In contrast, Basak et al. [53] performed a different approach for the mechanical testing and finally reported that the composite material was soft with low strength. El Bahy et al. [57] showed that a higher ratio of PVA presented a higher tensile strength and Eb. Furthermore, Jain et al. [54] reported that a high ratio of PVA to a lower concentration of gelatin could increase the TS of fabricated film. Lastly, Khan et al. [56] reported that the highest TS is at 100 Krad but with the lowest PVA concentration of 5%. The Eb of the highest content of PVA will be the lowest percentage. Besides, Chaibi et al. [50] also reported that the microhardness of the gelatin-PVA film was different from the TS and Eb. The hardness (H) of the cross-linked gelatin was higher than the H of the modified gelatin (PVA).

### 4.3. Thermal Properties

The literature search showed that only Al-Mamun et al. [52], El-Kader et al. [58] and Khan et al. [56] mentioned thermogravimetric analysis and thermal properties. Al-Mamun et al. [52] scrutinised that the elevated irradiation doses on the fabricated films demonstrated a low weight loss percentage (%). El-Kader et al. [58] reported that with the increase of PVA content in the blended samples, the thermal stability of the samples increases. Besides, Khan et al. [56] described that the blended and irradiated samples showed higher thermal stability than the pure and non-irradiated samples, respectively.

### 4.4. Swelling Study

As for crosslinking approach by gamma-irradiation, only a few articles reported this parameter. Al-Mamun et al. [52] reported that for the first 15 min, the non-irradiated film showed a higher water uptake than the irradiated films. However, after 7 days, the highest dose of the irradiated film retains most of the water content. These findings were supported by Ismaiel et al. [51] that stated increasing the gelatin ratio demonstrated a higher percentage of swelling properties. However, increasing the dosage of irradiation will decrease the swelling percentage. In addition, Jain et al. [54] unravelled no significant difference with various amounts of gelatin. The unesterified insert, however, swells faster but started deforming and disintegrating after 60 min.

### 4.5. Differential Scanning Calorimetry and UV-vis Analysis

Three articles reported the DSC analysis with a crosslinker while another two studies did not use any crosslinker. Al-Mamun et al. [52] reported no significant changes between different doses with the two peaks formed. Meanwhile, Chaibi et al. [50] presented that adding any other plasticising or crosslinker will induce crystallinity of the gelatin-based films. El-Kader et al. [58] showed that the DSC thermograms only disclose one single peak of the blended films while the other two articles reported the UV-vis analysis, one with a crosslinker and another without a crosslinker. Ismaiel et al. [51] reported that a high dose of irradiation improves the transparency of the polymer blend. Additionally, El-Kader et al. [58] demonstrated that the blended composition highly affected the polymer structure by changing the absorption spectra.

### 4.6. Molecular Structure

Four articles reported the XRD analysis including glutaraldehyde crosslinking, gamma-irradiation crosslinking and another two studies without a crosslinker. Using samples crosslinked with glutaraldehyde, Basak et al. [53] reported that all of the products were well crystallised. In samples crosslinked by gamma-irradiation, Ebnalwaled et al. [55] reported that distortion of the crystalline structure occurs with the increase of both gelatin and irradiation doses. As for the non-crosslinking approach, El-Kader et al. [58] claimed that the 1:1 blended sample shows that the crystals formed in the PVA did not prevent the compatibility between the amorphous regions of the homopolymers.

### 4.7. Crosslinking Degree and Surface Hydrophilicity

Chaibi et al. [50] reported that without glutaraldehyde, the *Nε* would be slightly decreased. But with the presence of glutaraldehyde, *Nε* showed a further decrease. Other studies did not include this parameter, especially for the studies using gamma-irradiation to crosslink gelatin and PVA. Basak et al. [53] demonstrated that 44° contact angle of the film with no crosslinking approach has been used in their experiment. 

### 4.8. In Vitro Biocompatibility and Cytotoxicity

Basak et al. [53] reported that the composite film is highly compatible with human blood. They reported that the cell viability is more than 90.14% and is less toxic. Besides, A-Mamun et al. [52] showed no significant changes along with the different gamma irradiation doses in terms of the number of cell death. However, there was no cell death in the non-irradiated films.

## 5. Discussion for Each Characterisation Parameters

There are two main parts that we are focusing on in this review, which are the fabrication methods and the characteristics of the gelatin-PVA thin film. Our review demonstrates that fabricated film from gelatin and PVA mixture could be a promising 3D design and suitable for medical applications, especially in TE and pharmaceuticals. 

### 5.1. Fabrication of Gelatin-PVA Film by Film Casting Method

Previous studies showed various ratios on the combination of gelatin to PVA in fabricating the blended films. Table 1 shows that most studies utilised a 1:1 ratio of gelatin to PVA. However, the original concentrations of each material used by different researchers are varied while some researchers omit that information from their studies. This has rendered the estimation of a reliable film ratio as the initial concentration is not similar. To put things into perspective, the ratio of the gelatin to PVA added to fabricate the film depends on the desired specifications, and characterised by appropriate parameters. All of the mixed solutions in all of the studies containing either gelatin-PVA only or with other plasticisers or crosslinkers are solution-casted onto a plate or a mould and let to dry. This is probably the best and easiest method to fabricate a thin film. However, based on the studies, there are different ways of drying the film. Some of the methods require a high temperature to improve the drying of the films. The use of an oven at 37 °C and 50 °C for 24 h may improve the drying of the films. Several other methods require airflow but only at room temperature through a laminar flow or an ambient airflow. Most of the methods dry the films at room temperature but require a longer time up to 6 days. Drying the film higher than the room temperature may decrease the drying time and thus, is the best method to be applied. However, films dried at high temperatures may affect the functionality of the film [59], especially the protein-based compositions. The detailed method might have some differences, but all studies use the casting method as a fabrication method of gelatin-PVA film.

Some of the films were added with crosslinkers to improve the linkage of the two different substances such as gelatin and PVA. Crosslinkers help improve the mechanical characteristics while avoiding cell-mediated contractions [60]. Crosslinking is a direct way of modifying the mechanical, biological and degrading properties of a scaffold. It is defined as the induction of chemical or physical linkages among the polymer chains [61]. Most of the studies above choose glutaraldehyde as the crosslinker. Glutaraldehyde can modify amines to form an intermediate resulting in the process of polymerisation [62]. However, glutaraldehyde with a high concentration is quite toxic to the cells and can cause cell death [63]. Gamma-irradiation is also another method that can improve the linkage between gelatin and PVA. Another alternative to prevent damage towards the scaffold or the film itself is the use of genipin, which is biocompatible and a well-known crosslinker. When compared to aldehyde glues, it has been discovered to cause less cytotoxicity and inflammatory responses (formaldehyde and glutaraldehyde) [64,65].

### 5.2. Characterisation of the Film

The SEM analysis showed that several studies that performed a higher concentration of gelatin to PVA ratio may improve the film’s surface, making it smooth and uniformly homogenise. A higher dose of gamma irradiation may cause the roughness of the film’s surface hence increasing the crosslinking between gelatin and PVA. However, some studies claimed to conclude that gamma irradiation did not impart any changes in the morphology of the films. The AFM analysis performed by Jain et al. [54] showed a smooth, homogenous and uniform surface. This indicates the homogenous reaction between gelatin and PVA. Therefore, gamma-irradiation would be a better choice and method to crosslink gelatin and PVA compared to using glutaraldehyde that is highly toxic towards cells at a particular concentration [66,67].

**Figure 8:**
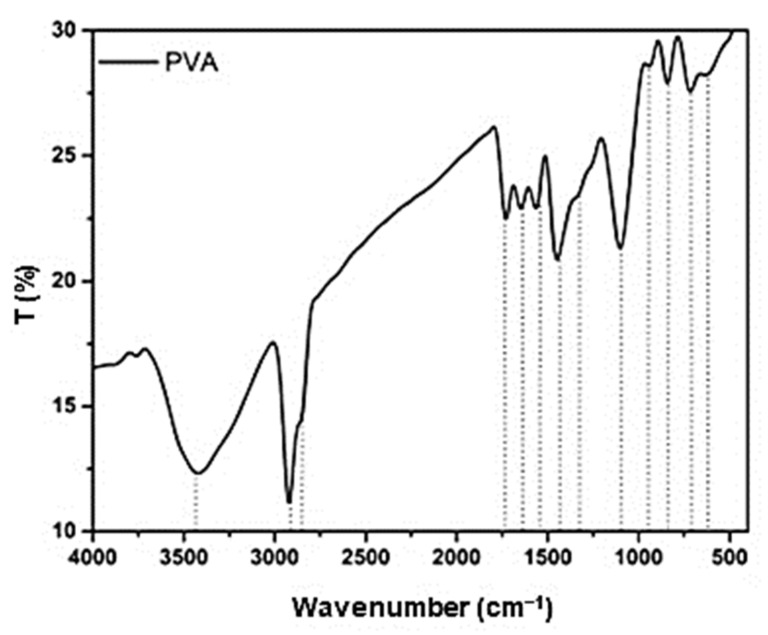
FTIR result for PVA [69]. Used under the Creative Commons License (http://creativecommons.org/licenses/by/4.0/ accessed on 1 August 2021).

**Figure 9:**
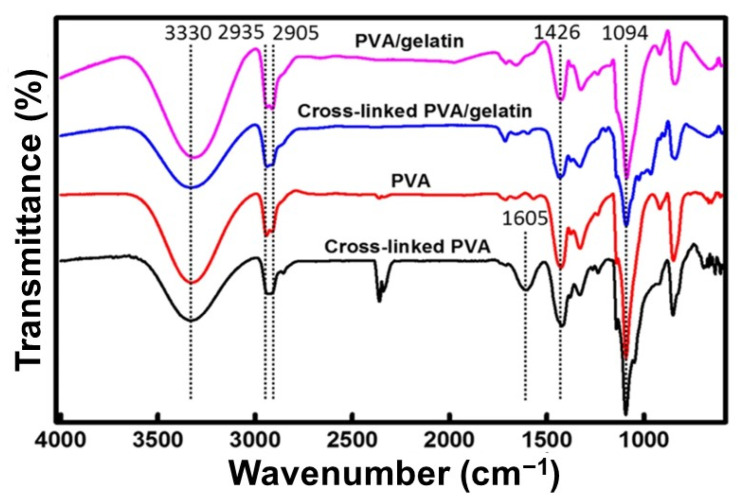
FTIR result for Gelatin and PVA [70]. Used under the Creative Commons License (http://creativecommons.org/licenses/by/4.0/).

FTIR analysis helps to analyse the functional group in a mixture. The films crosslinked with gamma irradiation showed a positive peak in which this prominent peak shows that crosslinking method improves the chemical interaction between gelatin and PVA. As for glutaraldehyde, Basak et al. [53] did not perform a comparison to a control group without a crosslinking method. However, the group revealed a prominent peak with the mixture of gelatin, PVA and HAP. Chaibi et al. [50] reported that the addition of glutaraldehyde proved the chemical interaction between gelatin and PVA. For the non-crosslinked films, El Bahy et al. [57] reported an absence of chemical interactions while other articles reported an occurrence of chemical interactions. These might be the reasons for the different concentrations of gelatin and PVA or without any plasticisers that can help in strengthening the bonding [68]. To support the study, Figure 8 shows a recent study for the FTIR analysis of PVA which shows a peak at 2926 cm^−1^, 2845 cm^−1^ and 1710 cm^−1^ indicating the stretching vibrations of methyl groups, methylene band and acetyl carbonyl groups in PVA structure. Another reference can be observed from Figure 9 where it shows a difference in peak between a crosslinked gelatin and PVA with a crosslinker or without a crosslinker indicating the existence of intermolecular and intramolecular hydrogen bonds.

Mechanical properties are essential for the handling and durability of the films. One of the methods for testing a film’s mechanical properties is by using a machine that is capable to measure the elongation at break, tensile strength and their Young’s modulus (stiffness) by pulling the film at different ends simultaneously. Some studies from the selected articles declared to use machine or techniques such as computer-controlled Universal Testing Machine, Hounsfield Series S Testing Machine and Shore durometer. As stated in the findings, most of the studies reported that a higher concentration of PVA may increase the TS of the film and the percentage of Eb. The properties of PVA may cause this due to its excellent mechanical properties [71]. However, increasing the gamma radiation dose will decrease the tensile strength of the film. This is because a high irradiation dose might decrease the functionality of the film and its functional group thus breaking the chemical bonds. Other than that, thermal stability is an important parameter for the stability of the sample for storage and applications. It is usually defined as the capabilities of one polymer to withstand heat and maintain its elasticity, toughness and strength at different temperature properties [72]. Thermal stability depends on the structure, bond lengths, bond angle and electronegativity of the compounds. Based on the three articles, a higher dose of radiation or merely just the radiation and the increase or addition of PVA content may improve the thermal stability. As a result, the higher thermal stability observed for TGA and its derivative blended samples was due to intermolecular cross-linking reaction, which resulted in highly compatible impactful blended polymers. 

As for DSC analysis, the two peaks formed in the study by Al-Mamun et al. [52] indicated the melting of gelatin and PVA. Sobral et al. [73] suggested that the polymers were not thermodynamically miscible in the mixture and that emerging phase separation was introduced during the DSC analysis. According to the different values of αDSC, it is evident that adding an agent (PVA or glutaraldehyde) to the gelatin will induce a decrease in the crystallinity of the gelatin-based films. By disrupting the crystallisation process, the plasticiser and/or crosslinking agent may affect the microstructure of the gelatin matrix. This actively demonstrates that crosslinked samples tend to have a very small degree of crystallinity. The swelling study reported by the three articles showed similar and rational results. Ismaiel et al. [51] stated that a higher irradiation dose decreases the swelling percentage at 20 kGy with a minimum value of 5 kGy. Al-Mamun et al. [52] stated that an irradiation dose at 3.5 kGy might increase the swelling and this is supported by Ismaiel et al. The ratio of gelatin as documented by Jain et al. [54] showed no significant difference, which is contradicting a report by Ismaiel et al. This may be caused by the crosslinking reaction by gamma irradiation [51]. As for UV-vis analysis, based on two articles, it has been shown that blending of gelatin and PVA affects the absorption spectra and improves the transparency with a higher irradiation dosage.

The contact angle of the films was only reported by Basak et al. [53] in which the films reported providing a hydrophilicity property at less than 90° which concludes a good wetting and hydrophilic surface that provides a suitable cell attachment [74]. Differences in the surface energy regulate cell attachment whereby higher energy hydrophilic surfaces facilitate adhesion while low surface energy substrates prevent cell adhesion [75]. This indicates that the prepared membranes would be more stable and beneficial for cell growth. There are other reports that mentioned PVA has low protein affinities that lead to the limitation of cell binding or cell attachment and will appear as a rounded shape. However, mixing with gelatin improved the cell attachment and cell adhesion due to the arginine-glycine-aspartic acid integrin-binding sequence of gelatin with the involvement of Aα-chain and Bβ-chain [70]. Thangprasert et al. also reported that cells such as osteoblast adhered, aggregated and elongated on the walls of the pores of a gelatin-PVA scaffold. This shows a great cell attachment and cell proliferation, suitable for any kind of cell depending on the application [76]. As for XRD analysis, all samples show crystallinity. However, increasing the dose of gamma-irradiation and gelatin content may distort the crystalline structure. This may be due to the molecular chain crosslinking, which transforms regularly arranged crystallites into non-arranged crystallites by forming new bonds between the neighbouring chains [77]. Any change in the peak intensity after irradiation indicates that the materials’ structure has changed significantly [78]. Only Chaibi et al. [50] reported on the crosslinking degree. The slight decrease in *Nε* without glutaraldehyde indicates physical crosslinking of the blend. The decrease in *Nε* with glutaraldehyde indicates that glutaraldehyde introduces new groups involved in chemical crosslinking. 

**Figure 10:**
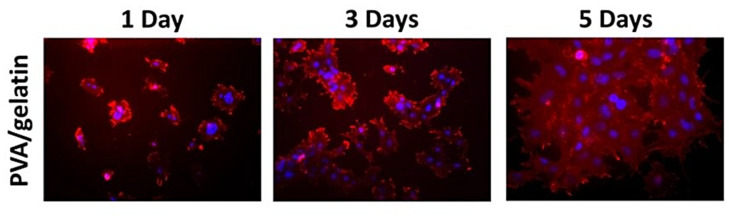
Fluorescence images of proliferation of fibroblasts cells [70]. Used under the Creative Commons License (http://creativecommons.org/licenses/by/4.0/).

In vitro evaluation of gelatin-PVA scaffold specifically for films is under research. The only biocompatibility and cell viability tests were reported by Basak et al. [53] whereby the film was described as highly haemocompatible. Besides, it is suitable for cell viability proving the absence of toxicity of the films towards cells. Al-Mamun et al. [52] stated that non-irradiated films did not show cell death in an in vitro cytotoxicity test. It is to note that it was a different method of cytotoxicity test whereby they used shrimp lethality bioassay method, which is different compared to the cytotoxicity test on human cells. However, the reports concluded that the death of the shrimp was caused by the formation of a viscous layer of PVA that inhibits the oxygen permeability [79]. There are many studies that reported both PVA and gelatin are non-toxic and exhibit high cell viability with high cell proliferation when seeded on gelatin-PVA scaffolds [70,76]. Biocompatibility test is very crucial and should be studied in the biomaterial field to know the ability and functionality of a fabricated scaffold, especially in TE and regenerative medicine. To support this review, a study by Huang et al., Figure 10, reported a good cell proliferation on a gelatin-PVA nanofibers, proving good biocompatibility and less toxicity of the biomaterial gelatin and PVA towards the cell. Regarding in vivo studies of gelatin-PVA film, there is a lack of studies focused on this aspect. However, to understand more regarding its efficacy by using this material, there was an in vivo study using gelatin-PVA scaffold in the form of a hydrogel. Using Wistar rats, the wound closure took 12 days to reach 95% wound contraction by identifying the wound closure and its histological analysis. The hydrogel showed no toxicity and offers adequate handling qualities and hydrophilicity, making it easier to attach to the wound bed. Furthermore, newly generated tissues were not damaged during the process of extracting hydrogels from the wounds in order to take images, and the hydrogels also were not clung to the wound bed [80]. 

## 6. Challenges and Limitations of Gelatin-PVA Film for Commercial Applications

TE aims to resemble natural processes and many attempts are being undertaken to construct nearly every tissue and organ in the human body via in vitro study. Further study is required to address many of the remaining restrictions in the fabrication process despite the recent advances in scaffold mechanical characteristics, porosity and bioactivity. The fabrication of these types of bioscaffolds can be a challenge to the researchers as they need a controlled environment. The process of casting and air drying are both important to fabricate a blended film. One of the drawbacks of this fabrication technique is the time consumption for fabricating the films [81]. It may take up to one week to make it thoroughly dried. Not to mention air drying without a controlled environment may lead to differences in the different batches of the fabrication, which can be an issue for reproducibility. In addition, the process of dissolving the PVA took a long time depending on the degree of hydrolysis. A higher molecular weight needs to have a higher temperature compared to a lower molecular weight [82] leading to a higher cost due to the heating time. The overall obstacles in scaffold design and fabrication open the door to new and exciting application-oriented research in scaffold designs such as polymer assembly, surface topography, biocompatibility, biodegradability, mechanical properties, cell function and induced formation of natural tissue.

Commercially, the use of gelatin is commonly questioned for its original resources. Commercial gelatin sources are commonly from mammalian bone and hide, specifically bovine and porcine [83]. Due to the cultural and religious beliefs and consumers’ concerns, the gelatin could be rejected based on its origin. Other than that, a substantial amount of time is needed to fabricate the scaffold. Any proper immediate application of a scaffold is not feasible. Lastly, the bioscaffold cannot be stored for an extended period because the scaffold is prone to hydrolysis with continuous exposure to water activity due to the gelatin being hygroscopic.

## 7. Conclusions and Future Perspectives

This review has established that gelatin-PVA thin film is one of the biomaterial products that can be beneficial in TE and medical applications due to its potentials. Looking at previous research, it is possible to be applied in the future. It is the easiest method of fabrication and price-considerable materials. Besides, the currently available market supports the use of these materials although there are no final and best ratio for mixing of these two materials due to the differences in the study designs. Many researchers have reported the characteristics of the film, and the importance of fabricating and applying these composite biomaterials in the form of films in the medical industry has been proven due to the non-toxic properties towards humans. Moreover, the findings from this review may be a guide for future study in fabrication and characterising the gelatin-PVA thin film. In addition, it could be improved more based on the accumulated knowledge of this film development in the field of TE.

Based on the work presented by the researchers, many improvements could be done to explore more on this particular scaffold. One of the most important components in the triads of TE is the cell-to-scaffold interaction. More studies towards the application and the biocompatibility of the scaffold towards cells are needed in order to clarify the functionality and the capability of the scaffold to function as a reliable bioscaffold in the TE field. Furthermore, interactions between cells and scaffolds can directly impact cell adherence and morphology, which can alter a variety of biological processes. Physicochemical features have either direct or indirect impacts on these interactions; in other words, we are looking at the biocompatibility of a scaffold towards cells. Therefore, it is vital to perform in vitro testing at a lab-scale or production stage before in vivo, pre-clinical or clinical stage in the future.

## Figures and Tables

**Table 1:** Concentration, ratio, fabrication methods of the film and molecular weight of PVA.

No	Author	Concentration of Gelatin	Concentration of PVA	Ratio (Gel:PVA)	Crosslinker	Method of Fabrication	Molecular Weight of PVA
1	Al-Mamun et al. (2020) [52]	0.11 g/mL	0.11 g/mL	1:1	Gamma-irradiation	Mixed by solution. Blend at pH 2, cast on silicon cloths followed by ambient air drying to form final films. Crosslinked by gamma-irradiation at different dosages for 1 h.	70,000 g/mol
2	Ismaiel et al. (2018) [51]	6 g	4 g	1:3 1:1 3:1	Gamma-irradiation	Mixed by powder into 125 mL of water. Then 0.5 mL citric acid is added and mixed for 90 min before casting into a plexiglass plate. Blends dried at 500 °C in oven for 12 h. The films were then heated in thermosetting oven at 950 °C for 1 h to induce crosslinking reaction.	450,000 g/mol
3	Basak et al. (2018) [53]	5%	5%	1:2	Glutaraldehyde	Film mixed by solution. Added with 200 mg HAP in 10 mL acetic acid. 2% glutaraldehyde added dropwise. Solution then casted at 40 °C for 24 h.	115,000 kDa
4	Ebnalwaled et al. (2017) [55]	Not stated	Not stated	1:1	Gamma-irradiation	PVA and gelatin powder were added in 125 mL water, stirred at 90 °C for 30 min. Then 0.5 mL of citric acid 0.1 N (plasticiser) was added and mixed. Solution added with copper oxide nanoparticles and mixed at room temperature for 30 min. Homogenised solution casted on glass plate and dried at room temperature. Films were then gamma irradiated at different dose.	450,000 g/mol
5	Khan et al. (2010) [56]	Not stated	Not stated	100:0 95:5 90:10 85:15	Gamma-irradiation	Mixed by solution at different ratio. Mixed solution is casted onto silicon paper covered glass plate and dried at room temperature for 48 h. Dried films were cut and stored in laminated polythene bag and kept in desiccators in room temperature. Upon testing, films were irradiated under gamma radiation at different doses.	Not stated
6	El Bahy et al. (2012) [57]	Not stated	Not stated	1:9 1:4 3:7 2:3 1:1 3:2 7:3 4:1 9:1	None	Mixed by solution at different ratio. Casted on polyethylene plates and dried at room temperature. Films were then placed in desiccator containing silica gel.	15,000 g/mol
7	Jain et al. (2011) [54]	1, 2, 3%	10%	1:10 1:5 3:10	None	Mixing by solution at different ratios. 500 uL of glycerol (plasticiser) added and mixed together. Esterification between PVA and gelatin initiated by adding 50–100 uL HCl and blend. Ciprofloxacin hydrochloride was added to the blend under magnetic stirring for homogenous dispersion. Casted on Petri plates and dried at room temperature in laminar flow. Dried film rinsed with sodium hydroxide solution.	125,000 g
8	El-Kader et al. (2010) [58]	Not stated	Not stated	1:9 3:7 4:6 1:1 7:3	None	Mixed by solutions at different ratio at 50 °C. Blends casted onto stainless steel Petri dishes, dried at room temperature for 6 days.	125, 000 g
9	Chaibi et al. (2015) [50]	5 g in 100 mL	0.2% by weight	29:43	Glutaraldehyde	Mixed by solution. 15 mL of blend casted in polystyrene Petri dish. 0.1 mm thickness film obtained. Crosslinked with different concentration of glutaraldehyde via post-crosslinking method. Films soaked in 15 mL of glutaraldehyde for 24 h.	70,000–100,000 g/mol

**Table 2:** Characterisation of gelatin-PVA film.

No	Author	Parameters	Result	Conclusion
1	Al-Mamun et al. (2020) [52]	FTIR spectroscopy Thermogravimetric analysis DSC analysis Tensile properties Water uptake SEM analysis In vitro cytotoxicity study	FTIR: Higher doses of irradiation shows prominent peaks than lower doses. TGA: Degradation or weight loss% decreased along the increasing of irradiation doses. DSC: No significant changes between different doses. 2 peaks formed, showing the melting of gelatin and PVA. Mechanical properties: TS increasing with the increasing radiation doses showing the increase in crosslinking. No significant difference between non-crosslinked. Eb% decreasing with the increasing radiation doses. Higher Em value from the highest dose of radiation. Water uptake: First 15 min, non-irradiated films uptake more water than irradiated films. After 7 days, the highest dose of radiated film retains most amount of water. SEM analysis: No significant changes in the surface morphology. In vitro cytotoxicity study: No significant changes, number of cell death varies. No trend along the increasing of doses. But no death in non-irradiated films.	Gamma irradiated films (crosslinked) can be potential non-toxic material with increased thermal and mechanical properties along with good water-retention capacity to be used as artificial articular cartilage.
2	Ismaiel et al. (2018) [51]	FTIR spectroscopy Swelling study SEM analysis UV-vis	FTIR spectroscopy: Irradiated films shows prominent peak than non-irradiated films. Swelling study: Higher gelatin ratio shows higher percentage, increasing the dose of irradiation will decrease the percentage of swelling. SEM analysis: Radius of the circles on the surface decreased as the concentration of gelatin increased. Gamma-irradiated films, no circular shape observed. Increasing the dose of gamma causes cracking on the films. UV-vis: High dose of irradiation improves the transparency of polymer blend indicating improvement in miscibility.	Exposure of gamma irradiation can be used as a technique to improve the miscibility of polymer blend. Increasing gelatin content into polymer blends increases the swelling ratios due to hydrophilicity of PVA.
3	Basak et al. (2018) [53]	FTIR spectroscopy Mechanical testing SEM analysis XRD analysis Cell viability Contact angle Biocompatibility test	FTIR spectroscopy: Interfacial interaction between PVA-Gelatin/HAP composites confirmed. Mechanical testing: Shore D measurement scale shows 14, composite material is very soft in nature SEM analysis: Presence of HAP within the polymer matrix is clearly indicated. Spherical shape of HAP presence XRD analysis: Product was well crystallised. Cell viability: >90.14% viability and has less toxic effect. Contact angle: 44 which indicates the biocompatible property of composite material Hemocompatibility test: Composite film highly compatible with human blood.	XRD result confirms the synthesized powder was nearly pure HAP. Typical absorption of HAP in the scaffolds observed by FTIR. Haemolysis and viability test show the developed composite is highly haemocompatible and the contact angle shows its hydrophilic nature. It could be applied in in vivo study. This composite material could be applied in bone tissue engineering application in future.
4	Ebnalwaled et al. (2017) [55]	FTIR spectroscopy SEM analysis XRD analysis	FTIR spectroscopy: Increasing gelatin concentration, vibrations band shifted to higher wavenumber and intensities decreased. SEM analysis: Whitening of composite increased by increasing of gelatin. Gamma dose increased; surface roughness increased. XRD analysis: Increasing of full width at half maximum (FWHM) as the gelatin ratio increased, same manner as irradiation doses increased. It indicates the distortion of the crystalline structure due to crosslinking.	The blend films exhibit highest absorption coefficient in the UV region, can act as UV shielding films. FTIR, XRD and SEM shows positive interaction between polymer blends and CuO nanoparticles. Gamma irradiation led to increase in absorption coefficient, refractive index and optical conductivity.
5	Chaibi et al. (2015) [50]	FTIR spectroscopy DSC analysis Microhardness Crosslinking degree	FTIR analysis: Physical crosslinking bands between gelatin and PVA shown. Addition of glutaraldehyde in the blend proved the chemical crosslinking. DSC analysis: Adding any agent will induce crystallinity of gelatin-based films. Microhardness: The hardness (H) of the crosslinked gelatin is higher than the H of the modified gelatin (PVA). Crosslinking degree: Without GTA, *Nε* will slightly decrease. With GTA, the *Nε* will further decrease showing the crosslinking between gelatin and other plasticisers.	Crosslinked gelatin films with plasticiser exhibit an improved mechanical behaviour. Gelatin established a physical crosslinking reaction with the GLY/PVA mixture, in the absence of GTA. Present of GTA leads to chemical crosslinking reaction with gelatin. The *Nε* decreases even more with the blends of gelatin with Gly/PVA crosslinked with GTA.
6	El Bahy et al. (2012) [57]	FTIR spectroscopy Tensile strength Elongation at break	FTIR analysis: No new bands formed, no chemical interaction between gelatin and PVA. Tensile strength: Higher ratio of PVA, higher tensile strength of blend. Elongation at break: Higher ratio of PVA, higher elongation at break point.	For FTIR analysis, interaction between the two polymers are only physical and not chemical. No esterification. The hydrogen bonding may rearrange the chains in gelatin in a certain manner. It improves TS and Eb.
7	Jain et al. (2011) [54]	FTIR spectroscopy Mechanical properties Swelling study SEM analysis AFM analysis Wettability (contact angle)	FTIR spectroscopy: Complete esterification of carboxylic acid of gelatin with PVA. Mechanical properties: Esterified inserts shows higher mechanical properties compared to unesterified inserts. Swelling study: No significant difference with different amount of gelatin. Unesterified insert swells faster but started deforming and disintegrating after 60 min. SEM and AFM analysis: No phase separation observed indicating good compatibility between matrix and drug. Contact angle: <50, hydrophilic nature of PVA and gelatin. Good contact with ocular mucosa.	Ciprofloxacin-loaded PVA–gelatin inserts had smooth and homogeneous surfaces, high light transmittance, hydrophilicity and superior mechanical and mucoadhesive properties.
8	El-Kader et al. (2010) [58]	Thermogravimetric analysis DSC analysis X-ray diffraction UV-vis analysis	TGA analysis: Thermal stability increased with the increasing of PVA content in blended samples. DSC analysis: DSC thermograms of the blended samples showed one single peak. XRD analysis: 1:1 blended sample shows crystals formed in PVA did not prevent the compatibility between the amorphous regions of homopolymers. UV-vis analysis: Blend composition highly affected the polymer structure	All blended samples data showed single-phase behaviour. Blend composition affects polymer structure. Higher PVA content shows higher thermal stability, 1:1 ratio indicates no crystals formed in PVA and did not prevent compatibility between amorphous regions of homopolymers.
9	Khan et al. (2010) [56]	FTIR analysis Thermal property Tensile properties Morphological characteristic	FTIR analysis: Grafted film does not show any characteristic peak corresponding to carbonyl group and amino group indicating the crosslinking through this group. Thermal properties: The glass point of blended film with 5% PVA and irradiated is higher, temperature for weight loss is also higher. Tensile properties: Highest TS at 100 Krad but with the lowest concentration of PVA (5%). Elongation at break of the highest content of PVA will decrease. SEM analysis: In the untreated pure gelatin, there were some unbound micro granules. However, irradiated blend shows some interaction between gelatin and PVA due to irradiation.	Gelatin-PVA blends modified with gamma radiation improves mechanical and thermal properties of gelatin films.

## Data Availability

The data presented in this study are available on request from the corresponding author.
